# Malaria Parasite cGMP-dependent Protein Kinase Regulates Blood Stage Merozoite Secretory Organelle Discharge and Egress

**DOI:** 10.1371/journal.ppat.1003344

**Published:** 2013-05-09

**Authors:** Christine R. Collins, Fiona Hackett, Malcolm Strath, Maria Penzo, Chrislaine Withers-Martinez, David A. Baker, Michael J. Blackman

**Affiliations:** 1 Division of Parasitology, MRC National Institute for Medical Research, Mill Hill, London, United Kingdom; 2 Faculty of Infectious and Tropical Diseases, London School of Hygiene and Tropical Medicine, London, United Kingdom; Washington University School of Medicine, United States of America

## Abstract

The malaria parasite replicates within an intraerythrocytic parasitophorous vacuole (PV). Eventually, in a tightly regulated process called egress, proteins of the PV and intracellular merozoite surface are modified by an essential parasite serine protease called PfSUB1, whilst the enclosing PV and erythrocyte membranes rupture, releasing merozoites to invade fresh erythrocytes. Inhibition of the *Plasmodium falciparum* cGMP-dependent protein kinase (PfPKG) prevents egress, but the underlying mechanism is unknown. Here we show that PfPKG activity is required for PfSUB1 discharge into the PV, as well as for release of distinct merozoite organelles called micronemes. Stimulation of PfPKG by inhibiting parasite phosphodiesterase activity induces premature PfSUB1 discharge and egress of developmentally immature, non-invasive parasites. Our findings identify the signalling pathway that regulates PfSUB1 function and egress, and raise the possibility of targeting PfPKG or parasite phosphodiesterases in therapeutic approaches to dysregulate critical protease-mediated steps in the parasite life cycle.

## Author Summary

Malaria is a scourge of the developing world and many researchers are seeking new ways to treat and control the disease. Malaria is caused by a single-celled parasite that grows within red blood cells, eventually rupturing them to release invasive merozoites in a process known as egress. In earlier work we found that, just prior to egress, an enzyme called SUB1 is released from the intracellular parasites into the vacuole in which they reside. SUB1 then cleaves a number of proteins required for egress and development of invasive merozoites. The signals that control SUB1 discharge are poorly understood. In this work, we show that SUB1 release requires the activity of another parasite enzyme called protein kinase G (PKG), which is in turn activated by a small molecule called cGMP. Inhibition of PKG blocks SUB1 discharge and egress, whilst premature activation of PKG by a member of a class of compounds called phosphodiesterase inhibitors, which increase cGMP levels in the parasite, induces premature egress of mostly non-invasive merozoites. These findings increase our understanding of egress and show that both malarial PKG and parasite phosphodiesterases (which are validated drug targets in humans) are potential targets for a new class of antimalarial drugs.

## Introduction

Clinical malaria results from replication of asexual forms of the malaria parasite in red blood cells (RBC). At the end of each intraerythrocytic replication cycle, the infected RBC ruptures, allowing egress of merozoites which invade fresh cells. Egress is sensitive to certain protease inhibitors, and a number of studies have implicated serine [1] or cysteine [2], [3] proteases in the process. Previous work has shown that the developing intracellular parasite expresses a subtilisin-like serine protease called SUB1, which is initially stored in specialised secretory organelles called exonemes [1]. Just prior to egress, SUB1 is discharged into the lumen of the parasitophorous vacuole (PV), the intraerythrocytic compartment in which the dividing parasite resides. Once in the PV, SUB1 precisely cleaves a number of important parasite proteins. In the case of the most virulent malaria pathogen *Plasmodium falciparum*, SUB1 substrates include the merozoite surface proteins MSP1, MSP6, MSP7, and MSRP2, the papain-like putative PV proteases SERA5 and SERA6, and the rhoptry protein RAP1 [1], [4], [5]. Cleavage of the SERA proteins may convert them to active enzymes that function in egress [6]. Pharmacological inhibition of *P. falciparum* SUB1 (PfSUB1; PlasmoDB ID PF3D7_0507500) prevents egress or results in release of non-invasive merozoites [1], [3], [4], suggesting that some or all of the proteolytic events mediated by SUB1 are important for PV membrane (PVM) or RBC membrane rupture, or merozoite maturation.

The malaria parasite replicates by schizogony, in which up to 5 cycles of nuclear division produce a multinucleated schizont bounded by a single plasma membrane, before cytokinesis eventually allows budding (segmentation) of daughter merozoites. Because of this mode of replication, it has long been speculated that strict temporal regulation of egress must be critical, since premature egress would release immature merozoites. This has promoted interest in the signalling pathways that govern egress, and recent work has implicated two parasite kinases. Knockdown of the *P. falciparum* calcium-dependent kinase CDPK5 produces a block in egress [7], whilst treatment of parasites with the trisubstituted pyrrole 4-[2-(4-fluorophenyl)-5-(1-methylpiperidine-4-yl)-1*H*-pyrrol-3-yl] pyridine (compound 1; C1), a reversible but potent and selective inhibitor of the parasite guanosine 3′ 5′-cyclic monophosphate (cGMP)-dependent protein kinase (PKG) (PlasmoDB ID PF3D7_1436600) [8], [9], results in accumulation of mature segmented schizonts which similarly do not undergo egress [10]. Whilst mechanical rupture of the mature CDPK5-depleted schizonts released invasive merozoites, similar rupture of C1-treated parasites released non-invasive merozoites, indicating that *P. falciparum* CDPK5 and PKG (PfPKG) act at different stages of egress, or in distinct pathways [7]. However, neither the functional role of these kinases in egress, nor the relationship between their activity and the protease-mediated mechanisms operating at egress, is known.

We have used pharmacological tools and an inhibitor-resistant *P. falciparum* mutant to examine the role of PfPKG in egress. We show that PfPKG activity is required for discharge of PfSUB1 into the PV, implicating PfPKG as a key upstream regulator of PfSUB1 activity against endogenous substrates. Dysregulation of the PfPKG-mediated pathway results in either a block in egress or premature release of predominantly immature, non-invasive merozoites.

## Results

### Two structurally distinct inhibitors of PfPKG block proteolytic processing of PfSUB1 substrates but do not inhibit PfSUB1 catalytic activity

**Figure 1:**
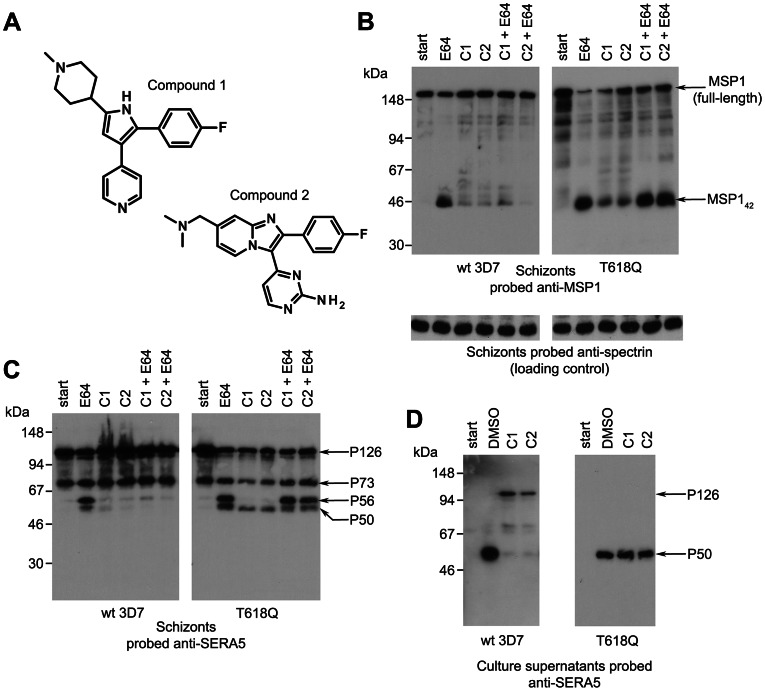
Structurally distinct PfPKG inhibitors block PfSUB1-mediated proteolytic processing in *P. falciparum*. (A) Molecular structures of C1 and C2. (B–D) Mature wild-type (wt 3D7) or PfPKG_T618Q_ *P. falciparum* schizonts were supplemented with cysteine protease inhibitor E64 only (50 µM), C1 only (2.5 µM), C2 only (1.5 µM), or combinations indicated, or vehicle only (DMSO, 1% v/v). Cultures were sampled immediately (start) or after further incubation for 4 h. SDS extracts of the residual schizonts (B, C) or culture supernatants (D) were analysed by Western blot, probing with mAbs specific for MSP1 or SERA5. An antibody to erythrocyte spectrin was used as loading control for the schizont extracts. Processing of MSP1 by PfSUB1 converts it to 4 polypeptides that include the MSP1_42_ fragment [4]. In wt parasites, but not in the PfPKG_T618Q_ clone, both C1 and C2 inhibited PfSUB1-mediated conversion of MSP1 to MSP1_42_ (B) or conversion of the SERA5 precursor P126 to the processing products P56 and P50 (C), although some background processing is evidence in the presence of the inhibitors, perhaps due to imperfect synchrony of the parasite cultures. The relatively low abundance of processed SERA5 in the PfPKG_T618Q_ schizont extracts in the presence of C1 or C2 only (C) are likely due to their loss from the schizonts at egress. Note that E64 inhibits conversion of P56 to P50, which is not mediated by PfSUB1 [1]. In (D), treatment of wt 3D7 with C1 or C2 resulted in release of only low levels of unprocessed SERA5 P126, whereas the normally-processed P50 form appeared in the PfPKG_T618Q_ supernatants irrespective of the presence of the PfPKG inhibitors. See also Figure S1 in Text S1.

Processing of MSP1 by PfSUB1 comprises precise cleavage at three known positions to convert the MSP1 precursor to four major polypeptide products that include a ∼42 kDa species called MSP1_42_ [4], [5]. PfSUB1-mediated processing of the abundant soluble PV protein SERA5 involves cleavage of the ∼126 kDa precursor (P126) at two defined sites to release a central domain called P56 (which is subsequently converted by a distinct, unknown protease to a smaller P50 form) [1]. We previously noticed [7] that treatment of schizonts with C1 (Figure 1A) prevents PfSUB1-mediated proteolytic processing of MSP1. To further investigate this finding, we examined the effects of C1 as well as a second, structurally-dissimilar PfPKG inhibitor, (4-[7-[(dimethylamino)methyl]-2-(4-fluorphenyl)imidazo[1,2-*α*]pyridine-3-yl]pyrimidin-2-amine (compound 2; C2, Figure 1A) [11], on processing of both MSP1 and SERA5. Like C1, C2 prevents egress [10]. As a control compound for these assays we used the cysteine protease inhibitor E64. Like C1 and C2, E64 prevents schizont rupture (e.g. [12]); however, it does not inhibit PfSUB1 activity [5] and it does not affect merozoite maturation or the invasive capacity of the ‘trapped’ merozoites [12], [13]. As shown in Figure 1B–C (blots marked ‘wt 3D7’), both C1 and C2 either alone or in combination with E64, but not E64 alone, efficiently inhibited PfSUB1-mediated processing of MSP1 and SERA5 in schizonts. This result showed that two independent inhibitors of PfPKG selectively interfere with proteolytic processing of two distinct PfSUB1 substrates.

To seek further support for this conclusion, we examined the effect of the compounds on the release of PfSUB1 substrates into culture medium. Upon normal egress, both SERA5 and MSP1 (which is eventually shed from the surface of free merozoites [14]) are released into the surrounding medium. Because E64 blocks egress, treatment with E64 was expected to reduce loss of merozoite surface-bound MSP1 from schizonts (due to egress) irrespective of the effects of C1 and C2. However, in the final stages of schizont maturation, the PVM and RBC membrane become permeabilized, allowing the partial release of components of the PV into the external milieu [15]; this is unaffected by the presence of E64 [15]. We therefore expected that some release of SERA5 from mature schizonts into culture supernatants might occur irrespective of the presence of reagents that block egress. As shown in Figure 1D (wt 3D7 blot), limited release of SERA5 into culture supernatants did occur in cultures treated with C1 or C2, but this protein was observed mostly in unprocessed form, in contrast to the exclusively processed SERA5 found in control supernatants. This result was consistent with those from analysis of the schizont extracts, confirming that both C1 and C2 prevent PfSUB1-mediated processing of two distinct protein substrates.

To confirm the selectivity of the impact of C1 and C2 on proteolytic processing of PfSUB1 substrates in the parasite, we took advantage of a *P. falciparum* mutant in which the *pfpkg* gene has been modified to substitute the small ‘gatekeeper’ residue, Thr618, with a bulkier Gln residue [16]. Based on modelling studies and analogies with PKG of the related apicomplexan *Toxoplasma*, Thr618 is predicted to lie close to the ATP binding pocket in PfPKG [9]. Whilst its substitution with Gln does not significantly affect access of ATP to the binding pocket, it prevents access of C1 and C2 to an adjoining pocket. As a result, PfPKG_T618Q_ parasites are selectively resistant to both C1 and C2 [10], [16]. We reasoned that if the inhibitory effects of C1 and C2 on PfSUB1-mediated processing were a direct result of PfPKG inhibition, as opposed to an off-target effect, C1 and C2 would not affect processing of PfSUB1 substrates in the PfPKG_T618Q_ clone. As shown in Figure 1B-D (blots labelled ‘T618Q’), this was the case; at concentrations that efficiently block processing in wt 3D7, neither C1 nor C2 had any effect on PfSUB1-mediated processing in the PfPKG_T618Q_ parasites.

Aside from its modified *pfpkg* locus, the PfPKG_T618Q_ parasite clone is isogenic with wt 3D7 and expresses wild-type PfSUB1. The lack of inhibition of PfSUB1-mediated processing in the PfPKG_T618Q_ parasites by either C1 or C2 therefore suggested that the PfPKG inhibitors do not directly affect PfSUB1 catalytic activity. In support of this, our earlier study [7] showed that C1 does not inhibit the activity of recombinant PfSUB1 (rPfSUB1). However, to assess the possibility that either compound might modulate the catalytic activity of the parasite enzyme, we examined the effects of both compounds on the activity of native PfSUB1, which can be specifically assayed [17] in detergent extracts of wt parasites. As shown in Figure S1 in Text S1, at concentrations of up to 20 µM (i.e. at least 8-fold higher than those concentrations that completely block egress), neither C1 nor C2 had any effect on parasite-derived PfSUB1 hydrolytic activity *in vitro* against a synthetic peptide substrate based on a PfSUB1 cleavage site within SERA5 [1]. These results show that the observed effect of C1 and C2 on processing of PfSUB1 substrates in the parasite is not a result of inhibition of PfSUB1 catalytic activity. Collectively, our findings show that the inhibition of PfSUB1-mediated processing by these two structurally distinct PfPKG inhibitors in wt 3D7 parasites is not an off-target effect, but is a direct consequence of their inhibition of PfPKG kinase activity.

### Biosynthesis, maturation and trafficking to exonemes of PfSUB1 occurs late in *P. falciparum* schizont maturation and is unaffected by PfPKG inhibitors

**Figure 2:**
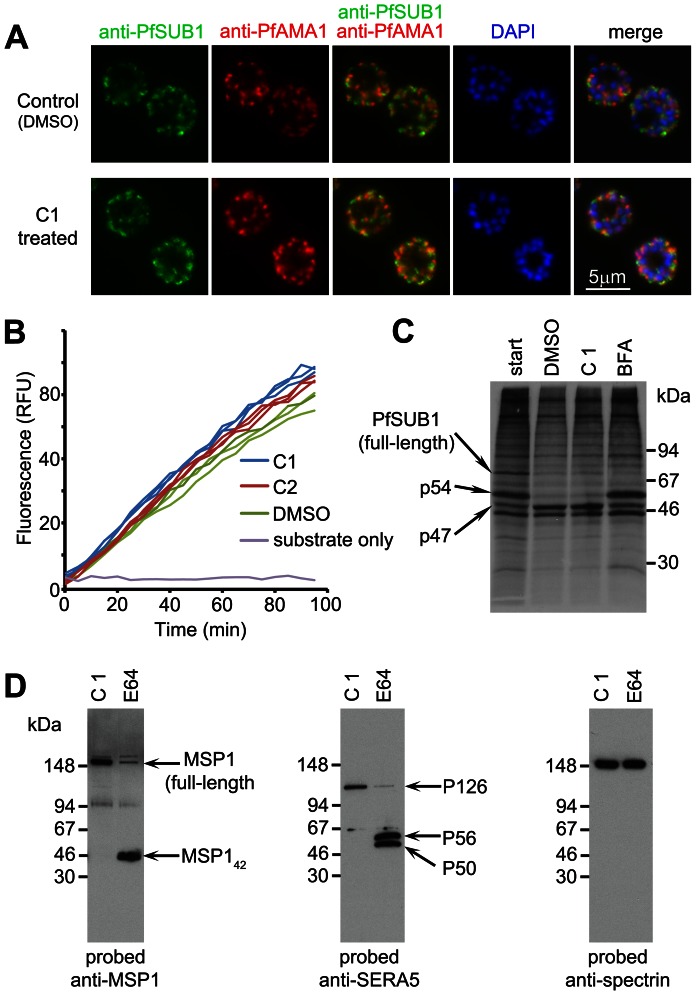
C1 and C2 do not affect expression, trafficking or maturation of PfSUB1. (A) IFA of mature wt 3D7 parasites following treatment for 6 h with DMSO only (1% v/v) or 2.5 µM C1, probed with the anti-PfSUB1 mAb NIMP.M7 or a rabbit anti-PfAMA1 serum. The PfPKG inhibitor had no effect on localisation or expression of PfSUB1 or PfAMA1. For clarity, the anti-PfSUB1 and anti-PfAMA1 signals are shown merged in addition to a final merge with the DAPI signal. Similar results were obtained following treatment with 1.5 µM C2 (not shown). Note that treatment with the PKG inhibitors also had no effect on schizont replication as determined by counts of the average number of nuclei per schizont (data not shown, but compare DAPI staining of untreated and treated schizonts). (B) Progress curves showing cleavage *in vitro* of fluorogenic PfSUB1 substrate SERA5st1F-6R by extracts of wt 3D7 schizonts produced following culture for 6 h in the presence of C1 or C2, or DMSO only (control). Identities of the extracts used (tested in triplicate) are colour coded. (C) Maturation of PfSUB1 is unaffected by C1. Schizonts biosynthetically pulse-radiolabeled with [^35^S]-methionine/cysteine were further cultured for 1 h in medium containing DMSO only (control), C1 (2.5 µM), or brefeldin A (BFA; 10 µg/ml). Schizonts were then harvested and PfSUB1 immunoprecipitated with a rabbit anti-PfSUB1 antibody and visualised by SDS PAGE and fluorography. Positions of the full-length (∼82 kDa) PfSUB1 primary translation product, as well as the p54 and p47 maturation products, are arrowed. As expected, BFA inhibited conversion of p54 to p47 as this occurs in a parasite Golgi or post-Golgi compartment [40]. C1 had no effect on PfSUB1 maturation. The intense band at ∼45 kDa in all tracks is not derived from PfSUB1, but is non-specifically bound by the antibodies [40]. (D) Processing of endogenous PfSUB1 substrates following release of a C1-mediated egress block. Schizonts cultured for 7 h in the presence of C1 were washed and incubated for a further 30 min in medium containing C1 or E64 only, then examined by Western blot. An antibody to RBC spectrin was used as a loading control. See also Figure S2 in Text S1, and Movies S1 and S2.

PfSUB1 is synthesised as a zymogen which undergoes proteolytic maturation [17] then is stored in exonemes until its discharge into the PV. The above-described experiments with C1 and C2 involved only short-term treatment (up to 4 h) of mature schizonts, so we considered it unlikely that their inhibitory effects on PfSUB1-mediated processing could be due to interference with biosynthesis and trafficking of PfSUB1 to exonemes. Indeed, previous work has shown that even prolonged (up to 24 h) treatment of schizonts with C1 does not affect localisation of a range of merozoite proteins, including proteins of the micronemes, a distinct set of secretory organelles that play a primary role in invasion [10]. However, to address this possibility we examined the effects of C1 and C2 on targeting of PfSUB1 to exonemes. For this, we first produced a panel of 4 monoclonal antibodies (mAbs) all reactive with the catalytic domain of PfSUB1 (Figure S2 in Text S1). Using these mAbs, indirect immunofluorescence analysis (IFA) of developmental stages spanning the asexual life cycle showed that PfSUB1 expression is restricted to the late stages of schizont maturation, being first detectable at the 13–14 nuclei stage (∼40 h post-invasion in the 3D7 clone, which has an asexual blood-stage cycle in our laboratory of 45–46 h) (Figure S2D in Text S1). As expected, based on previous studies using parasites expressing epitope-tagged PfSUB1 [1], the PfSUB1 signal was distinct from that of the microneme protein PfAMA1 (Figure S3E in Text S1). To examine the effects of the PfPKG inhibitors on trafficking of PfSUB1, wt 3D7 schizonts at the 10–12 nuclei stage (i.e. just before the appearance of detectable PfSUB1 expression) were supplemented with C1 or C2, cultured further for 5 h until mature but not yet undergoing egress, then examined by IFA with the anti-PfSUB1 mAb NIMP.M9. The PfPKG inhibitors had no effect on the pattern or intensity of the PfSUB1-specific signal (Figure 2A), suggesting that they do not affect PfSUB1 expression or its trafficking to exonemes. In agreement with previous observations [10], the treatment also had no effect on trafficking of PfAMA1 to micronemes. Equal numbers of schizonts from control cultures or cultures treated with C1 or C2 were then extracted, and levels of endogenous PfSUB1 in the extracts quantified *in vitro*. There was no decrease in PfSUB1 activity in extracts of the C1 or C2-treated parasites (Figure 2B), indicating that the PfPKG inhibitors do not impair intracellular accumulation of active PfSUB1. To further examine the effects of PfPKG inhibition on the fate of PfSUB1, pulse-chase experiments were performed in the presence of C1. No effects on PfSUB1 maturation were detected (Figure 2C).

Both C1 and C2 are competitive, reversible inhibitors of apicomplexan PKG [8], [11]. We reasoned that, if the PfPKG inhibitors did not perturb trafficking of PfSUB1 to exonemes, schizonts allowed to develop to full maturity (i.e. up to or beyond the point at which they would normally have egressed) in the presence of a PfPKG inhibitor should possess functional exonemes containing enzymatically active PfSUB1. Simply washing the PfPKG inhibitor away from such fully mature schizonts should reverse the egress block, allowing discharge of PfSUB1 and subsequent proteolytic events. To test this prediction, purified schizonts at the 13–14 nuclei stage were supplemented with C1 or DMSO, then returned to culture and allowed to develop to the point where egress was almost complete in the control culture. At this point (∼7 h following C1 addition), the C1-containing culture contained virtually no free merozoites, indicative of the expected block in egress. The C1-stalled schizonts were then washed and further incubated in fresh medium containing C1 only (as a control), E64 only, or no additions. Time-lapse microscopic examination of the latter showed the predicted rapid egress of the stalled schizonts (Movie S1), with 85±6% of schizonts undergoing egress within 30 min of C1 removal (n = 3 independent experiments). This egress was efficiently prevented by the continued presence of C1 (not shown), or by E64 (Movie S2). Importantly, despite the block in egress exerted by E64, Western blot of the E64-treated schizonts just 30 min following removal of the C1 block showed that processing of the bulk of SERA5 and MSP1 had occurred within that short time-frame (Figure 2D), indicating rapid PfSUB1 activity upon C1 removal. This confirmed that, despite the potent egress block mediated by C1, the stalled schizonts are fully competent for exoneme discharge, PfSUB1-mediated proteolytic processing of endogenous substrates, and egress, all of which occurred rapidly in the minutes following inhibitor removal. Collectively, these results show that the inhibition of PfSUB1-mediated proteolysis of endogenous substrates observed upon treatment with the PfPKG inhibitors is not a result of a defect in PfSUB1 expression or maturation, exoneme biogenesis, or trafficking to exonemes.

### PfPKG inhibitors block discharge of exonemes and micronemes

**Figure 3:**
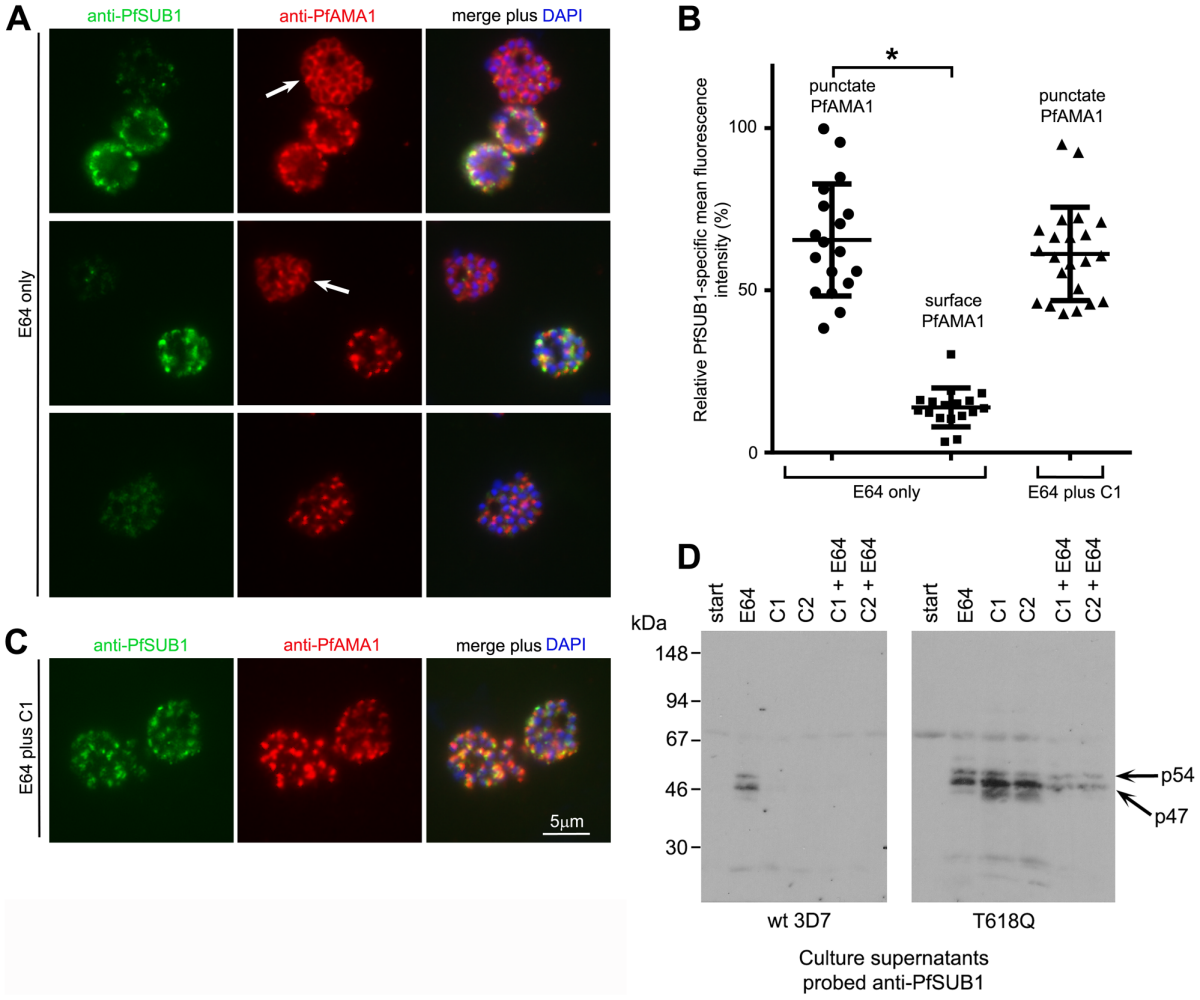
PfPKG inhibitors block discharge of exonemes and micronemes. (A) IFA of wt 3D7 parasites allowed to develop beyond the point of egress in the presence of E64 only (50 µM). Top two rows: segmented schizonts displaying a merozoite surface localisation of PfAMA1 (arrowed; ∼45% of the schizont population) as a result of its discharge from micronemes, exhibit a weak PfSUB1 signal compared to less mature schizonts in which both the PfAMA1 and PfSUB1 signals remain punctate. The bottom row of images in (A) shows a schizont in which the PfAMA1 signal is intermediate between punctate and merozoite surface. (B) Surface translocation of PfAMA1 is associated with loss or redistribution of the PfSUB1-specific IFA signal. The PfSUB1-specific mean fluorescence intensity in individual schizonts relative to that of the PfAMA1-specific signal was acquired as described in Materials and Methods. Individual relative fluorescence intensity values (at least 17 schizonts per group) are shown plotted, with mean and SD indicated. Within the parasites treated with E64 only, values were significantly lower for the schizonts displaying the merozoite surface PfAMA1 phenotype (Student's unpaired *t*-test, two-tailed P value<0.0001). There was no significant difference between the E64-treated and E64 plus C1-treated values for schizonts with the punctate PfAMA1 phenotype (see panel C). (C) IFA of wt 3D7 parasites allowed to develop beyond the point of egress in the presence of both E64 and C1. No discharge or relocalisation of PfSUB1 or PfAMA1 was evident in counts of >5,000 schizonts from a total of 3 independent experiments. Identical results were obtained with E64 plus C2 (not shown). (D) Western blot of culture supernatants from synchronous wt 3D7 or PfPKG_T618Q_ schizonts allowed to develop beyond the point of egress in the presence of E64 only (50 µM), C1 only (2.5 µM), C2 only (1.5 µM), or indicated combinations. Cultures were sampled immediately (start) or after incubation for 4 h. The p54 and p47 forms of PfSUB1 usually observed following its maturation are arrowed. The relatively low levels of PfSUB1 in supernatants of the PfPKG_T618Q_ parasites in the presence of C1 or C2 plus E64 are likely due to the block in egress mediated by E64. See also Figure S3 in Text S1.

Having ruled out the possibility that the PfPKG inhibitors interfere with PfSUB1 biosynthesis, maturation, trafficking, or catalytic activity, we hypothesised that their effects on proteolysis of endogenous PfSUB1 substrates might be due to a selective block in discharge of PfSUB1 from exonemes. To test this, we used IFA to examine the effects of C1 on the fate of PfSUB1 at egress. To allow a direct comparison between the contents of C1-stalled schizonts and control untreated schizonts, these experiments were again performed in the additional presence of E64 to prevent egress of those parasites not treated with C1. Antibodies to PfAMA1 were also included in the IFA analysis to detect any effects on microneme discharge. Highly mature schizonts approaching the point of egress were treated with either E64 alone or E64 plus C1. The parasites were then cultured for a further 2 h (during which time ∼50% of schizonts in control untreated cultures underwent egress), then finally fixed and analysed by IFA. Examination of the cultures treated with E64 alone showed that while ∼55% of the schizonts displayed the usual punctate micronemal PfAMA1 pattern, in the remaining ∼45% of the schizonts the anti-PfAMA1 signal took the form of a uniform fluorescence around the periphery of intraerythrocytic segmented merozoites (Figure 3A top 2 rows and Figure S3A in Text S1) consistent with release of the PfAMA1 onto the merozoite surface. This is well-documented to occur for PfAMA1 following normal egress, but has previously been only rarely observed in intracellular parasites e.g. [18], [19]. The high proportion of schizonts exhibiting merozoite surface PfAMA1 in these preparations therefore suggested that translocation onto the merozoite surface is more likely to occur when egress is prevented by E64. Remarkably, co-staining with the anti-PfSUB1 mAb NIMP.M7 showed that all schizonts exhibiting the merozoite surface PfAMA1 pattern (n>400, totalled from 3 independent experiments) were negative or only very weakly positive for PfSUB1, whilst the majority of those (presumably less mature) schizonts in which the PfAMA1 pattern remained punctate also retained their normal strong, punctate PfSUB1 IFA signal (Figure 3A top 2 rows and Figure S3A in Text S1). A quantitative analysis of the IFA signals associated with the different PfAMA1 phenotypes confirmed that schizonts with the merozoite surface PfAMA1 phenotype exhibited a significantly lower PfSUB1-specific mean fluorescence intensity (Figure 3B). The E64-treated schizonts - whether displaying a merozoite surface PfAMA1 pattern or not – were still retained within a bounding RBC membrane, as shown by IFA using anti-RBC antibodies, confirming the expected egress block (Figure S3B in Text S1). These observations suggested that, in intracellular parasites egress of which was blocked with E64, secretion of PfAMA1 onto the merozoite surface is associated with loss or extensive redistribution of PfSUB1. A small fraction (∼2%) of schizonts from the E64-treated cultures displayed a relatively weak and diffuse anti-PfSUB1 fluorescence signal but a still mostly punctate PfAMA1 signal (Figure 3A bottom row), suggesting that PfSUB1 can be discharged prior to PfAMA1 release.

In striking contrast to cultures treated with E64 only, schizonts treated with C1 plus E64 (or C1 only; not shown) exhibited uniformly strong, punctate PfAMA1 and PfSUB1 IFA signals (Figure 3C); examination of >5,000 schizonts from 3 independent experiments identified not a single C1-treated schizont in which any signs of PfAMA1 or PfSUB1 discharge were evident. Quantitative analysis found no significant difference in relative PfSUB1-specific mean fluorescence intensity between the C1 plus E64-treated schizonts and those treated with E64 only in which the PfAMA1 still appeared apically-located (i.e. not discharged) (Figure 3B; compare the two outer data columns on the graph). Confirmation that this apparent block in microneme and exoneme discharge was due to the action of C1 on PfPKG was obtained by repeating these experiments with the PfPKG_T618Q_ clone; as expected, in this case treatment with C1 plus E64 had no inhibitory effect on PfAMA1 discharge or loss of PfSUB1 (data not shown).

The apparent loss of PfSUB1 from wt schizonts in which discharge of PfAMA1 from micronemes had taken place, suggested that – despite the block in egress mediated by E64 and the retention of a bounding RBC membrane - the released PfSUB1 might be being lost into the surrounding medium, perhaps as a result of permeabilization of the PV and RBC membranes. In confirmation of this, PfSUB1 was detectable in culture supernatants of wt schizonts allowed to reach the point of egress in the presence of E64, and its release was efficiently blocked in schizonts cultured instead with C1 or C2 (Figure 3D). As expected, the PfPKG inhibitors did not prevent release of PfSUB1 from the PfPKG_T618Q_ parasite clone. Together with the IFA data, our results show that PfPKG activity is required for release of PfAMA1 from micronemes and discharge of PfSUB1 from exonemes. Since PfSUB1 discharge is essential for egress, this may fully explain the egress block mediated by the PfPKG inhibitors.

### The phosphodiesterase inhibitor zaprinast induces egress in a PfPKG-dependent manner

**Figure 4:**
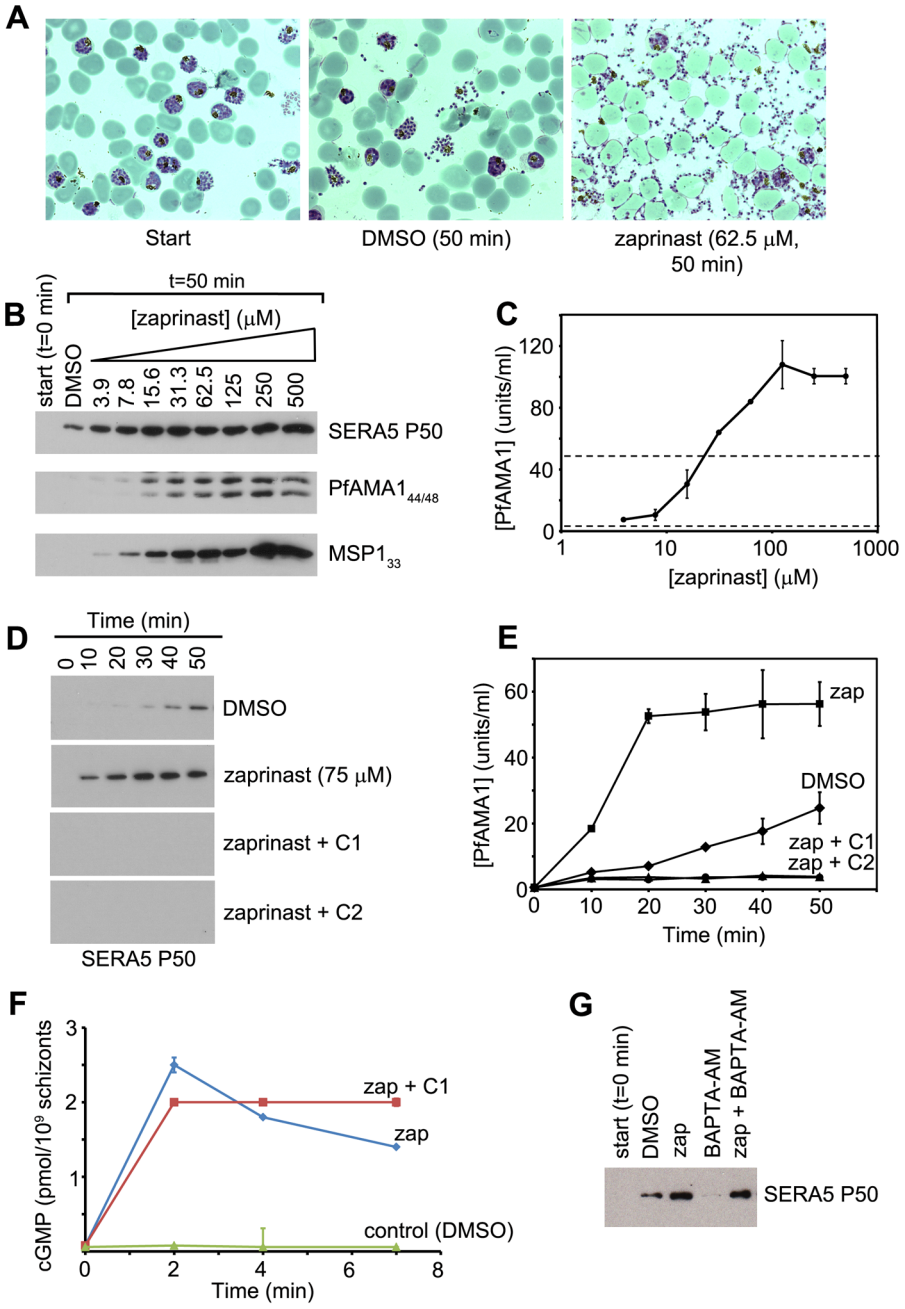
The phosphodiesterase inhibitor zaprinast induces premature egress in a cGMP-dependent manner. (A) Zaprinast induces merozoite egress. Mature schizonts (∼25% segmented) were supplemented with fresh RBCs to achieve a parasitaemia of ∼40%, then either processed at once for microscopic examination of Giemsa-stained thin films (Start), or after further culture for 50 min in the presence of zaprinast or vehicle only (DMSO, 0.5% v/v). (B) Dose-response profile of zaprinast-induced egress. Schizont preparations similar to those in (A), but in protein-free medium and without addition of uninfected RBCs, were incubated for 50 min in the presence of various concentrations of zaprinast. Culture supernatants were then examined by Western blot using mAbs reactive with SERA5, PfAMA1 or MSP1. (C) Quantitation by capture ELISA of shed PfAMA1, and its dependency on zaprinast concentration. Samples from cultures treated as in (B) for 50 min were serially diluted and assayed as described in Materials and Methods. Data are presented as mean values of triplicate readings ±SEM, with PfAMA1 concentrations in arbitrary units based on comparison with a standard. Dotted lines indicate [PfAMA1] values in the absence of zaprinast treatment (lower line), or 50% maximal levels (upper line), used to derive an ED_50_ for zaprinast of ∼25 µM. (D) Zaprinast-induced egress is blocked by PfPKG inhibitors. Western blot of supernatants harvested at different time-points from a schizont culture treated with a single concentration of zaprinast (75 µM), with or without the additional presence of C1 or C2. Blots were probed with the anti-SERA5 mAb 24C6.1F1. (E) Quantitation by ELISA of shed PfAMA1 levels in the supernatant samples probed in (D). Data are presented as in (C). (F) Zaprinast treatment increases cGMP levels in schizonts. The 0 min sample was taken just prior to addition of zaprinast ±C1. (G) BAPTA-AM inhibits egress, and this can be reversed by zaprinast treatment. Schizonts in protein-free medium were incubated in the presence of the indicated reagents for 50 min. BAPTA-AM was used at 100 µM, and zaprinast at 75 µM. See also Figure S4 in Text S1 and Movies S3 and S4.

Intracellular cGMP levels in eukaryotes are regulated by the interplay between cGMP synthesis and degradation, catalysed respectively by gyanylyl cyclases (GCs) and cyclic nucleotide phosphodiesterases (PDEs). The *P. falciparum* genome encodes just four putative PDEs, PfPDEα-δ [20], [21]. Recombinant PfPDEα, as well as the endogenous PDE activity found in extracts of asexual blood stage parasites, is sensitive to the PDE inhibitor zaprinast [22], with reported IC_50_ values of ∼4 µM [16], [20], [23]. In the light of our finding that inhibition of PfPKG prevents egress, we reasoned that pharmacological enhancement of steady-state cGMP levels by inhibiting PDE activity in mature schizonts might have the opposite effect and induce egress, by activating PfPKG. To test this, cultures enriched in mature but predominantly (∼75%) non-segmented wild-type 3D7 schizonts were incubated with a range of concentrations of zaprinast, then examined by time-lapse microscopy as well as by Giemsa stained thin films and Western blot analysis of culture supernatants. As shown in Movies S3 and S4, and Figure 4A–B, zaprinast induced rapid merozoite release, concomitant with the appearance in culture supernatants of high levels of processed forms of SERA5 and the shed forms of MSP1 and PfAMA1 normally released from merozoites following egress [19]. No full-length SERA5 was detected in the egress supernatants (Figure S4A in Text S1), indicative of normal PfSUB1 activity. Quantitation of the zaprinast-induced egress by enzyme-linked immunosorbent assay (ELISA) showed that levels of shed PfAMA1 in the supernatants of zaprinast-treated cultures were up to ∼25-fold higher than in control cultures after just 50 min of zaprinast treatment, and that the zaprinast concentration required for half-maximal induction of egress (ED_50_) was ∼25 µM (Figure 4C). Further experiments (Figure 4D–E) using a single concentration of zaprinast (75 µM; three-fold the ED_50_) to treat segmented schizonts showed that induction of egress as determined by an increase in supernatant SERA5 or PfAMA1 over control levels was detectable within 10 min of zaprinast addition, and peaked by 20–30 min. Importantly, the effects of zaprinast on wt schizonts were completely blocked in the presence of C1 or C2. Zaprinast also induced egress of the PfPKG_T618Q_ clone, but in this case C1 and C2 had no blocking effect (Figure S4B in Text S1). These results are consistent with the zaprinast-mediated induction of egress being due to stimulation of PfPKG by raising intracellular cGMP levels, as predicted, and not due to off-target effects. To confirm this, parasite cGMP levels were assayed following zaprinast treatment. This showed a rapid rise in schizont cGMP levels in the minutes immediately following zaprinast addition (Figure 4F); this also occurred in the presence of C1, showing that it was independent of PfPKG activity. Similar experiments found no change in schizont cAMP levels in the same period following zaprinast treatment (not shown), consistent with zaprinast inhibiting a predominantly cGMP-specific PDE activity in mature schizonts.

Egress of *Toxoplasma* can be induced by treatments that promote increases in cytosolic Ca^2+^, thought necessary and sufficient for microneme discharge and stimulation of parasite motility [24], [25]. As a result, induced *Toxoplasma* egress is sensitive to the intracellular calcium chelator BAPTA-AM, generally applied at extracellular concentrations ranging from 20–100 µM [26]. To examine the effects of BAPTA-AM on natural and zaprinast-induced *P. falciparum* egress, mature schizonts were treated with BAPTA-AM (100 µM) for 10 min at 21°C, then cultured further ±75 µM zaprinast, and levels of egress assessed by Western blot (Figure 4G). BAPTA-AM-treatment had a clear inhibitory effect on egress, suggesting that, as in *Toxoplasma*, malarial egress requires increases in cytosolic Ca^2+^ concentrations. The effect of BAPTA-AM was reversed by zaprinast (Figure 4G), perhaps suggesting that the stimulation of PfPKG induced by zaprinast leads to enhanced Ca^2+^ levels that effectively compensate for the effect of BAPTA-AM. Alternatively, PfPKG may induce secretion and egress in a calcium-independent manner.

### Zaprinast-induced egress involves rapid discharge of micronemes and exonemes

**Figure 5:**
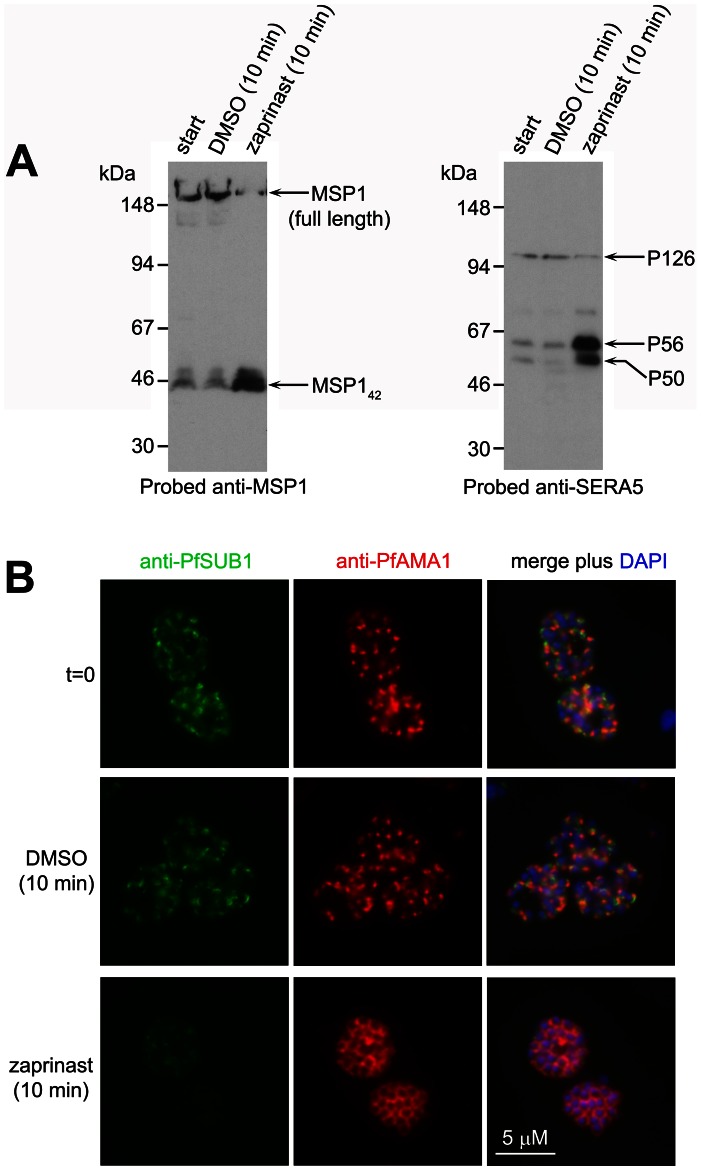
Zaprinast induces rapid discharge of exonemes and micronemes. (A) Zaprinast induces intracellular processing of MSP1 and SERA5. Western blot of schizonts treated as indicated in the additional presence of E64 to prevent egress. (B) Zaprinast treatment of schizonts (in the presence of E64) induces rapid relocalisation of PfAMA1 and loss of PfSUB1. Fixed, permeabilized schizonts were examined by IFA with the antibodies indicated. See also Figure S5 in Text S1.

To gain insight into the mechanisms underlying zaprinast-induced egress, we sought to directly visualise the effects of zaprinast on PfSUB1 localisation and activity against endogenous substrates in intact schizonts. Mature schizonts were treated for just 10 min with 75 µM zaprinast in the additional presence of E64 (to prevent egress, as described above), then the still intracellular parasites immediately fixed and examined by IFA, or analysed by Western blot. Zaprinast induced rapid intracellular processing of MSP1 and SERA5 (Figure 5A), concomitant with a dramatic relocalisation of the bulk of PfSUB1, as indicated by conversion from a bright punctate to a diffuse and/or weak IFA signal, and the discharge of PfAMA1 onto the surface of virtually all (98±1%) segmented schizonts (Figure 5B and Figure S5 in Text S1). Remarkably, zaprinast-induced relocalisation of PfSUB1 and PfAMA1 was evident even in immature (non-segmented) schizonts, but here took the form of translocation of both proteins to a striking circumferential punctate pattern, perhaps indicating accumulation of immature micronemes and exonemes at the periphery of the schizont (arrowed in Figure S5B in Text S1). Zaprinast-induced discharge of PfSUB1 and PfAMA1 as visualised by IFA was completely prevented by the addition of C1 just before zaprinast treatment (not shown). These results unambiguously demonstrate that PfSUB1 discharge can be induced by treatments expected to activate PfPKG.

### Zaprinast-induced egress results in premature release of developmentally immature, non-invasive merozoites

Microscopic comparisons (Figure S6 in Text S1) of the control and zaprinast-treated cultures from the experiments described in Figure 4 (n = 4) showed that zaprinast preferentially induced rupture of segmented schizonts. Despite this, and the large number of merozoites released, very few newly-invaded ‘ring’ stage forms were produced in the zaprinast-treated schizont cultures, indicating inefficient RBC invasion by the merozoites released upon zaprinast stimulation (see Figure 4A right-hand image and Figure S6 in Text S1). Normal egress is highly temporally regulated, and even in the most highly synchronised schizont populations we could produce using our standard protocols, egress of the entire population occurs only over a period of 3–4 hours. We speculated that the poor invasiveness of the merozoites released by zaprinast induction might be due to their enforced premature egress whilst developmentally immature. An alternative explanation was that zaprinast directly inhibits invasion by released merozoites. We reasoned that one way to distinguish between these possibilities would be to exploit the reversibility of the PfPKG inhibitors described above, and examine the invasive capacity of merozoites released upon zaprinast treatment of schizonts that had been allowed to develop to full maturity (i.e. up to or beyond the point at which they would normally have egressed) in the presence of C1.

**Figure 6:**
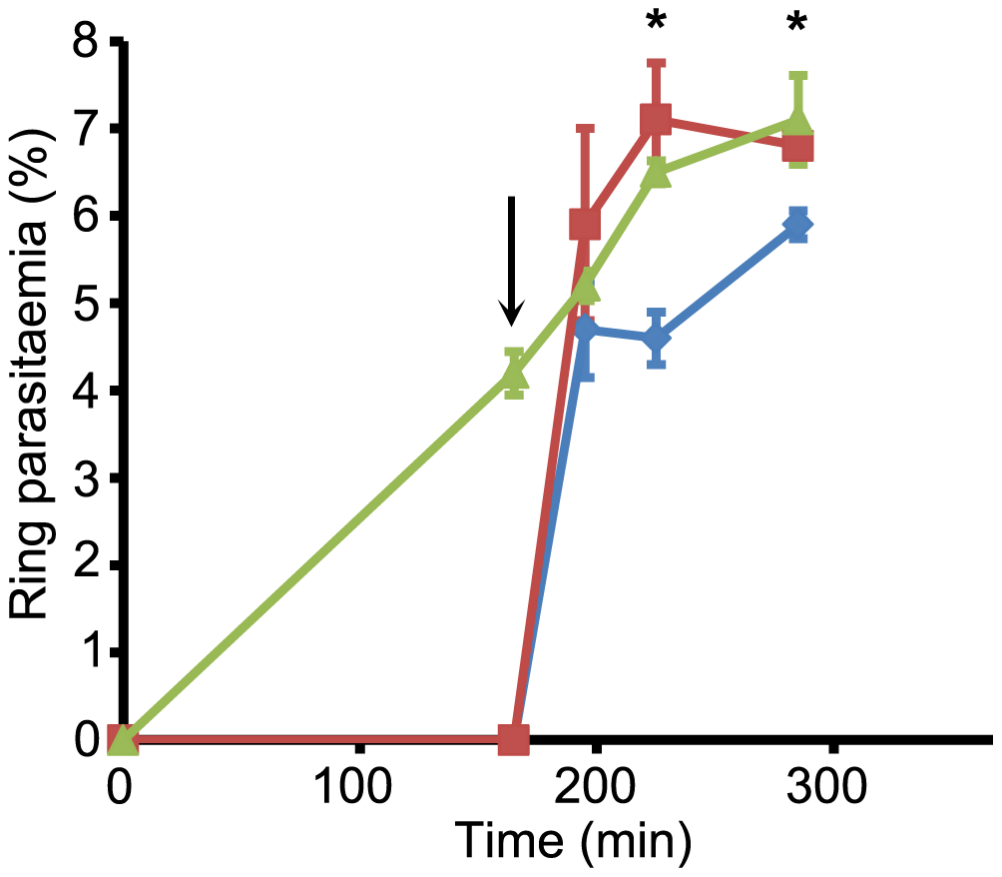
Zaprinast does not prevent invasion. Cultures containing mature schizonts (∼5% parasitaemia) were incubated in the absence (green) or presence (red and blue plots) of C1 (2.5 µM). After 160 min (arrowed), all cultures were washed and recultured in fresh medium minus C1, but with the addition of zaprinast (75 µM) to one culture (blue). Appearance of rings was monitored with time. Within 30 min following the wash and reculture step, the parasitaemia profiles had merged, demonstrating rapid invasion immediately following release of the C1 block, regardless of the presence of zaprinast. The subsequent relatively slow increase in parasitaemia in the zaprinast-containing culture (blue) likely reflects premature egress of the small number of residual immature schizonts still present at the point of reculture, which would release non-invasive merozoites. Time-points at which statistically different parasitaemia values were obtained for the two cultures which had been subjected to the C1 block are indicated with asterisks (Student's unpaired *t*-test, two-tailed P value = 0.0024 [225 min time-point] and P value = 0.0045 [285 min time-point]). Data are presented as mean values of triplicate counts ±SD. See also Figure S6 in Text S1.

To do this, a synchronous parasite culture (∼5% parasitaemia) containing mature schizonts was divided into three, supplemented with DMSO only (1 flask), or C1 (2 flasks) and allowed to develop for a further 4 h, at which point egress was well underway in the control (DMSO-treated) culture, as indicated by a >50% reduction in schizont numbers and the appearance of a substantial proportion of ring stage parasites (Figure 6). At this point, the C1-containing cultures contained no rings (<0.01%), due to the expected blockade of egress. All three cultures were then washed and re-cultured in fresh medium lacking C1. In the case of the previously C1-treated parasites, culture was continued in either the presence or absence of zaprinast. As shown in Figure 6, the ring parasitaemia of the C1-stalled cultures rose rapidly following removal of C1, reaching a value similar to that of the control culture within 30 min of the release of the egress block. Importantly, this occurred irrespective of the presence of zaprinast, proving that zaprinast at the concentration used (75 µM) does not inhibit invasion. These results support the evidence that the egress induced by zaprinast treatment of normal schizonts releases predominantly immature merozoites that lack the capacity to invade a new host RBC.

## Discussion

Three major conclusions can be drawn from our study, all of which advance our understanding of malarial egress. First, we have shown that the potent but fully reversible block in egress mediated by two selective inhibitors of PfPKG is accompanied by a corresponding block in release of exonemes and micronemes. This supports the model that discharge of PfSUB1 into the PV is a necessary step in egress. It also explains our earlier observation [7] that merozoites mechanically released from C1-stalled schizonts are non-invasive. These merozoites would not have been exposed to naturally-released PfSUB1, and in previous work we have shown that the formation of invasive merozoites requires PfSUB1-mediated proteolytic modification of merozoite surface proteins [4]. The egress block mediated by the PfPKG inhibitors could also be due to the inhibition of microneme discharge, since in *Toxoplasma* efficient egress requires release of the micronemal perforin-like protein TgPLP1, which plays a role in rupture of the PVM and host cell membrane [27]. There is no evidence for an analogous role of a perforin-like protein in *Plasmodium* asexual blood-stage egress, but this or another role for microneme proteins cannot be excluded. We were not overly surprised to observe inhibition of *P. falciparum* microneme discharge by the PfPKG inhibitors, since both C1 and C2 block microneme discharge in *Eimeria* sporozoites and *Toxoplasma* tachyzoites [11], [28]. Very recently, Lourido and colleagues [29] reported inhibition of *Toxoplasma* egress by PKG inhibitors and its stimulation by zaprinast, implicating the cGMP-dependent pathway in egress of coccidian parasites too. It is likely that analogous pathways operate in regulating organelle discharge in all apicomplexan parasites. However our data are the first to demonstrate the role of an apicomplexan PKG in regulating access of a key egress-related enzyme to its endogenous substrates.

**Figure 7:**
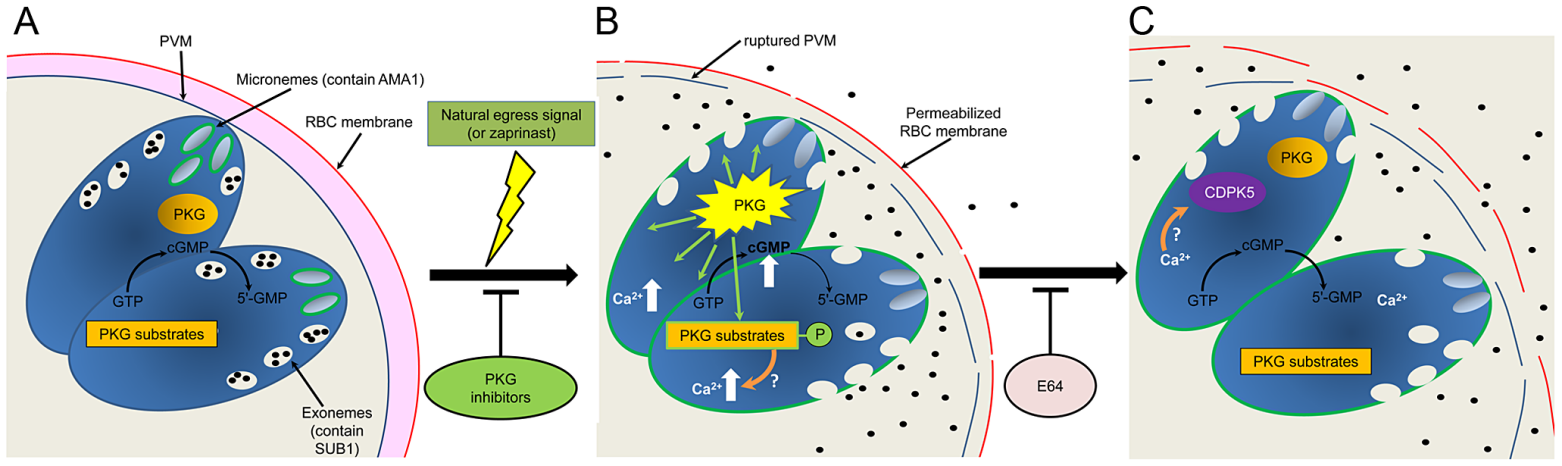
Model of the role of PKG in egress. (A) PfSUB1 and PfAMA1 are stored in exonemes and micronemes respectively in developing intracellular merozoites. Rapid turnover of cGMP maintains PfPKG in an ‘off’ state. (B) Upon an endogenous or exogenously-supplied signal (which can be mimicked by zaprinast-mediated inhibition of one or more parasite PDEs), cGMP levels are raised above threshold levels, resulting in activation of PfPKG and phosphorylation of its substrates. This leads to discharge of both PfSUB1 and PfAMA1, perhaps via an increase in cytosolic Ca^2+^ released from intracellular stores. PfAMA1 translocates onto the merozoite surface, whereas PfSUB1 is released into the PV. PVM rupture rapidly ensues, as well as limited permeabilization of the RBC membrane. (C) The Ca^2+^ flux may activate CDPK5 for a downstream role in egress. Eventual rupture of the RBC membrane is mediated by at least one E64-sensitive cysteine protease, allowing egress. PfPKG may rapidly return to an inactive state upon lowering of cGMP levels, whilst PfPKG substrates may be dephosphorylated through the action of endogenous phosphatases.

The second major conclusion of our study is that cGMP - through its capacity to activate PfPKG - is an endogenous regulator of malarial egress, and treatments that increase parasite cGMP levels induce premature egress in a PfPKG-dependent manner. Natural egress of *P. falciparum* schizonts is likely triggered by a signal that increases parasite cGMP levels. Zaprinast treatment presumably mimics that signal. This study did not extend to identifying the target of zaprinast in *P. falciparum*. However, the *pfpdeα* and *pfpdeδ* genes are non-essential in blood stages [20], [30], whilst the *pfpdeβ* gene is refractory to disruption (unpublished, D. Baker), indicating an indispensable role. PfPDEβ is therefore a good candidate for the target of zaprinast. Natural egress may be triggered by stimuli that down-regulate its activity, or by other signals that affect cGMP homeostasis (e.g. activation of guanylyl cyclase activity). Further work will be required to dissect this pathway, including the identification of PfPKG substrates. *Toxoplasma* egress involves mobilisation of Ca^2+^ from internal stores [24], [25]. Our findings that BAPTA-AM inhibits egress implies a similar calcium-dependence in *Plasmodium*. Indeed, during preparation of this manuscript, Agarwal et al. [31] reported that a sharp rise in intracellular Ca^2+^ is detectable in schizonts shortly before egress, and that treatment of schizonts with BAPTA-AM or a phospholipase C inhibitor (which likely inhibits Ca^2+^ release from intracellular stores) blocks egress, with a concomitant block in PfSUB1 discharge. A rise in intracellular Ca^2+^ just prior to egress of *P. falciparum* was also observed in a very recent study of Glushakova and colleagues [32]. The Ca^2+^ flux was unaffected by exogenous chelating agents in the culture medium, but was inhibited by BAPTA-AM, which also inhibited egress. In contrast, egress was accelerated by inhibitors of the parasite endoplasmic reticulum (ER) Ca^2+^-ATPase which pumps Ca^2+^ from the cytosol into the ER, as well as by the calcium ionophore A23187, further supporting the notion that the required Ca^2+^ is obtained from internal stores. Our present work showing that the BAPTA-AM-mediated egress block is overcome by zaprinast suggests that PfPKG acts to increase cytosolic Ca^2+^, or that it functions downstream or even independently of calcium in the regulatory pathway. The Ca^2+^ increase may be important for activation of CDPK5, the other *Plasmodium* kinase implicated in egress [7]. A simple model for the role of PKG in egress which may integrate our findings with these other strands of work is presented in Figure 7. Calcium signalling also regulates discharge of microneme proteins from extracellular merozoites [33], though how that is controlled independently of PfSUB1 discharge prior to egress is unknown.

The third conclusion from our work is that stringent timing of egress is critical for the asexual blood-stage parasite, since zaprinast-induced premature egress results in the release of largely non-invasive merozoites. This is in stark contrast to *Toxoplasma*, which replicates by endodyogeny, and where premature egress releases fully mature, invasive parasites. Indeed, this is thought to be advantageous for *Toxoplasma* survival *in vivo* as it provides a means for intracellular parasites to escape surveillance by cell-mediated immune cells [34]. The recent Glushakova *et al.* study [32] also found that egress could be induced by inhibitors of the parasite ER Ca^2+^-ATPase and A23187, although unfortunately those authors did not study the invasiveness of the released merozoites. Our results show that asexual blood-stage *P. falciparum* schizonts are egress-competent before the developing merozoites are invasion-competent. Furthermore, the dramatic alterations in exoneme and microneme localisation in even immature schizonts treated with zaprinast indicate that the parasites are sensitive to perturbation of cGMP homeostasis before segmentation. Previous studies reported that zaprinast prevents replication of *P. falciparum* [23], and our observations now provide an explanation for that. PfPKG is a druggable enzyme, and PDEs are validated chemotherapeutic targets, so our findings support efforts to exploit both PfPKG and parasite PDEs as antimalarial drug targets.

## Materials and Methods

### Reagents

Zaprinast, E64 and BAPTA-AM was from Sigma (UK). C1 (MRT00072273) and C2 (MRT00072329) were gifts of Medical Research Council Technology, London NW7 1AA. Fluorogenic PfSUB1 substrate SERA5st1F-6R (originally referred to as SERAst1F-6R), has been previously described [1], as has production of rPfSUB1 [17].

### Maintenance and synchronisation of *P. falciparum*

Asexual blood stages of *P. falciparum* were maintained and synchronized using standard procedures [1] in RPMI 1640 medium containing Albumax (Invitrogen). Mature schizonts were enriched from highly synchronous cultures using Percoll (GE Healthcare) as described previously [14].

### Antibodies and immunofluorescence

For IFA, air-dried thin films of parasite cultures were fixed in paraformaldehyde and permeabilized as described previously [6]. Slides were probed with the anti-PfSUB1 mAbs NIMP.M7, NIMP.M8, NIMP.M9 or NIMP.M10 (described here); the anti-SERA5 mAb 24C6.1F1 [35]; the human anti-MSP1 mAb X509 [36]; mouse and rabbit antisera specific for PfAMA1 [37], each diluted 1∶500; or a rabbit antiserum raised against human RBC ghosts (a kind gift of Tony Holder, NIMR), diluted 1∶1000. Secondary Alexafluor 488 or 594-conjugated antibodies specific for human, rabbit or mouse IgG (Invitrogen) were used at a dilution of 1∶1000. Samples were stained with 4,6-diamidino-2-phenylindol (DAPI) for nuclear staining then mounted in Citifluor (Citifluor Ltd., UK). Images were acquired using a Zeiss Axioplan 2 Imaging system (Carl Zeiss, Germany) and AxioVision 3.1 software. The anti-spectrin mAb SB-SP1 (α and β-specific) was purchased from Sigma. Western blots were prepared and probed as described previously [37].

Quantitation of IFA images was performed using Adobe Photoshop CS5 Extended (version 12.0.4) essentially as described by Bahtz et al. [38]. Briefly, defined areas of raw image files (TIFF format) exported from AxioVision 3.1, each corresponding to an individual schizont (mean pixel number ∼9,200 for each schizont) were selected with the Lasso Tool. The Histogram option was then used to obtain mean fluorescence intensity values of the PfSUB1-specific and PfAMA1-specific signals for each schizont. Background intensities were subtracted from each measurement, and then the relative intensity of the PfSUB1-specific signal for each schizont was calculated and expressed as a percentage using the following formula:
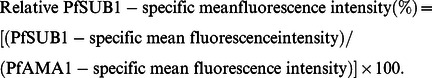
All compared images were taken during the same session, using equal and constant exposure times.

### Time-lapse video microscopy

Viewing chambers (internal volume ∼80 µl) for observation of live schizonts were constructed by adhering 22×64 mm borosilicate glass coverslips to microscope slides with strips of double-sided tape, leaving 4 mm gaps at each end. A schizont suspension in warm, gassed medium was introduced by capillary action into the pre-warmed chamber, the ends were sealed, and the slide transferred to a temperature-controlled microscope stage held at 37°C on an Axio Imager M1 microscope (Zeiss) equipped with an EC Plan-Neofluar 100×/1.3 oil immersion differential interference contrast (DIC) objective and an AxioCam MRm camera. Images were taken at 5 s intervals over a total of 30 min, then annotated and exported as QuickTime movies using Axiovision 3.1 software.

### Cyclic nucleotide assays and capture ELISA

To measure PfAMA1 released into parasite culture supernatants at egress, 96-well microtitre plates (Immulon, Nunc) were coated with anti-PfAMA1 mAb 4G2 [39] (2.5 µg/ml) in carbonate-bicarbonate buffer pH 9.6, then blocked with 1% (w/v) gelatin in PBS 0.25% (v/v) Tween 20. Serial dilutions of culture supernatants were applied to the plates. Following incubation for 1 h, plates were washed and a 1∶1000 dilution of rabbit anti-PfAMA1 serum applied. After a further 1 h incubation, this was replaced with an alkaline phosphatase-conjugated goat anti-rabbit IgG (Sigma), diluted 1∶30,000. After further incubation and washing, bound antibodies were detected with 4-nitrophenyl phosphate (Sigma) in diethanolamine buffer pH 9.8, measuring absorbance at 405 nm. Arithmetic PfAMA1 titres were expressed in arbitrary units/ml, by comparison with a standard.

For measurement of intracellular cyclic nucleotide levels, Percoll-enriched schizonts were suspended at a 5% haematocrit in gassed, protein-free RPMI 1640 at 37°C. Duplicate zero time samples (each containing 2×10^8^ schizonts) were pelleted by centrifugation (1 min, 13,000× g) and snap-frozen in a dry ice-ethanol mix. Further duplicate samples were then taken at intervals following addition of DMSO, zaprinast (75 µM) or zaprinast plus C1 (2.5 µM) and similarly processed. All samples were stored at −70°C until required for assay. cGMP and cAMP levels in schizont extracts were measured using a DetectX Direct cGMP or cAMP immunoassay kit (Arbor Assays, Michigan, USA), as directed by the manufacturer.

## Supporting Information

Movie S1 **Rapid egress of** ***P. falciparum*** **merozoites following release of a C1-mediated egress block.** Schizonts allowed to mature for 7 h in the presence of the PfPKG inhibitor C1 were washed, resuspended in fresh warm medium without C1 and immediately observed by time-lapse video microscopy. Virtually complete egress is observed within 30 min.
(MOV)

Movie S2 **Egress of** ***P. falciparum*** **merozoites following release of a C1-mediated egress block is prevented by the cysteine protease inhibitor E64.** Schizonts allowed to mature for 7 h in the presence of the PfPKG inhibitor C1 were washed, resuspended in fresh warm medium without C1 but containing E64 (50 µM) and immediately observed by time-lapse video microscopy. In contrast to the cultures lacking E43 (Movie S1) no egress takes place over 30 min.
(MOV)

Movie S3 **Egress in untreated mature schizonts.** Cultures of purified schizonts (∼25% segmented) were observed by time-lapse video microscopy for 30 min in the absence of zaprinast. Low levels of egress are evident.
(MOV)

Movie S4 **Zaprinast induces egress.** Cultures of purified schizonts (∼25% segmented) identical to those in Movie S3 were observed by time-lapse video microscopy for 30 min in the presence of zaprinast (100 µM). Substantially increased levels of egress are evident compared to Movie S3.
(MOV)

Text S1 **Supplemental Figures S1 to S6.**
(PDF)
