# Supplementary material for: Malaria Parasite cGMP-dependent Protein Kinase Regulates Blood Stage Merozoite Secretory Organelle Discharge and Egress
Source: PLoS Pathog. 2013 May 9;9(5):e1003344. doi: 10.1371/journal.ppat.1003344 (PMC3649973; doi:10.1371/journal.ppat.1003344)
Supplement: Text S1 — Supplemental Figures S1 to S6. (PDF) [file ppat.1003344.s005.pdf]

Supplemental figures and legends, movie legends and references

Malaria parasite cGMP-dependent protein kinase regulates blood stage merozoite secretory organelle discharge and egress

Christine R. Collins[1], Fiona Hackett[1], Malcolm Strath[1], Maria Penzo[1], Chrislaine Withers-Martinez[1], David A. Baker[2] and Michael J. Blackman[1*]

[1]Division of Parasitology, MRC National Institute for Medical Research, Mill Hill, London NW7 1AA, UK

[2]Faculty of Infectious and Tropical Diseases, London School of Hygiene and Tropical Medicine, London WC1E 7HT, UK

[*]Correspondence: mblackm@nimr.mrc.ac.uk. Tel. +44 208 816 2127. Fax.: +44 816 2730

Running title: *P. falciparum* PKG governs SUB1 release and egress

Supplemental figure legends

Figure S1. Compounds 1 and 2 do not inhibit the catalytic activity of parasite-derived PfSUB1

(A) PfSUB1 precisely cleaves its endogenous substrate SERA5 at two positions, referred to as site 1 and site 2 (Yeoh et al., 2007). A CHAPS extract of wt 3D7 parasites was supplemented with fluorogenic PfSUB1 substrate SERA5st1F-6R (0.2 μM final concentration), which is based on sequence flanking the site 1 processing site in SERA5 (Yeoh et al., 2007). Cleavage in the presence of C1 or C2 (20 μM final concentration of each, diluted 1:100 from a 2 mM stock in DMSO) was quantified by continuous measurement of the increase in fluorescence. Vehicle alone (DMSO, 1% v/v), or recombinant PfSUB1 prodomain (PD; 84 nM final concentration), a selective, nanomolar inhibitor of PfSUB1 (Jean et al., 2003), were used as negative and positive inhibitor controls respectively. The schizont extract activity was also completely sensitive to the selective peptidyl alpha-ketoamide PfSUB1 inhibitor KS-466 with an IC50 of ~1 μM, similar to that previously determined for rPfSUB1 (Withers-Martinez et al., 2012) (data not shown).

(B) Correct cleavage of the SERA5st1F-6R substrate by the schizont extracts was assessed by RP-HPLC analysis and detection of the fluorescent cleavage products, as described previously (Yeoh et al., 2007). SERA5st1F-6R is the N-acetylated peptide Ac-CIKAETEDDC, labelled on both Cys side-chains with tetramethylrhodamine (R). The two major cleavage products obtained following partial digestion of the substrate by the schizont extract were identical to those produced by rPfSUB1 (which cleaves only at the Glu-Thr bond within SERA5st1F-6R), confirming that essentially all cleavage of the substrate by the schizont extract was mediated by parasite-derived PfSUB1.

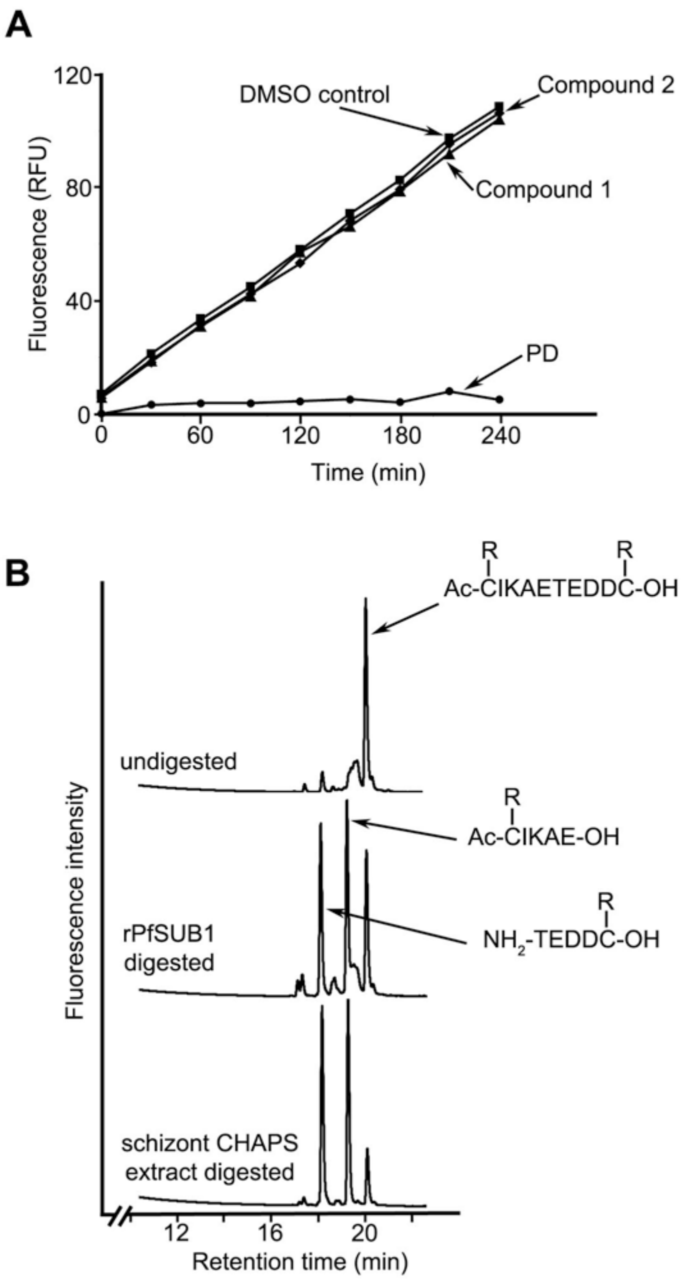

A
120
80
40
0
Fluorescence (RFU)
0
60
120
180
240
Time (min)
DMSO control
Compound 2
Compound 1
PD
B
Ac-CIKAETEDDC-OH
R
R
undigested
Ac-CIKAE-OH
R
rPfSUB1
digested
NH$_2$-TEDDC-OH
R
schizont CHAPS
extract digested
Fluorescence intensity
12
16
20
Retention time (min)

Figure S2. Anti-PfSUB1 monoclonal antibodies show that expression of PfSUB1 in *P. falciparum* exonemes is restricted to the late stages of asexual blood-stage schizogony

(A) Monoclonal antibodies (mAbs) were produced using splenic B cells obtained from BALB/c mice immunised with insect cell-derived rPfSUB1 (Withers-Martinez et al., 2002). The cells were fused with Sp2/0/Ag14 myeloma cells and cloned by limiting dilution using standard procedures, screening by dot blot against the immunising antigen for specific antibody production. SDS PAGE and Coomassie Blue staining analysis of immunoglobulin purified by Protein G Sepharose (GE Healthcare) affinity chromatography from culture supernatants of hybridoma clones NIMP.M7, NIMP.M8, NIMP.M9 and NIMP.M10. All four mAbs were subclass IgG1 as shown by Ouchterlony assay (data not shown), but displayed subtly distinct SDS PAGE profiles.

(B) Western blot analysis of rPfSUB1 and parasite schizont extracts, probed with each of the four mAbs or a rabbit anti-PfSUB1 antiserum (Blackman et al., 1998). Of the mAbs, only NIMP.M7 and NIMP.M8 showed any reactivity on Western blots, whether samples were chromatographed under reducing conditions (as here) or in the absence of reduction (not shown); both mAbs recognized both the p54 and p47 processed forms of PfSUB1.

(C) Pull-down assays. Control culture medium containing no IgG, or culture supernatants from an irrelevant (anti-SERA6) monoclonal hybridoma, or from hybridoma clones NIMP.M7, NIMP.M8, NIMP.M9, or NIMP.M10, were incubated with Protein G Sepharose beads in order to immobilise the IgG. The washed beads were then incubated with $rPfSUB1_{cat}$ (Withers-Martinez et al., 2012) for 30 min, then washed again. Bound $rPfSUB1_{cat}$ (arrowed) was detected by eluting bound proteins into SDS sample buffer and analysing the released proteins by Western blot, using the rabbit polyclonal anti-PfSUB1 antibody to probe the blots. The doublet visible at ~22 kDa in all pull-downs (arrowed) is due to Protein G leached from the beads, which binds to the HRP-conjugated anti-rabbit IgG antibodies used in the last step of the Western blot procedure. The extreme left-hand lane (labelled 'Total

(start)') is a sample of the starting rPfSUB1$_{cat}$ preparation used for all the pull-downs. U, unbound; B, bound to Protein G beads. All four mAbs bind rPfSUB1$_{cat}$ in solution.

(D) IFA of asexual blood-stage 3D7 parasites at a range of developmental stages, as indicated by the number of individual nuclei detected by DAPI staining. Expression of PfSUB1, as detected with monoclonal antibody NIMP.M7, first appears at the 13-14 nuclei stage. Identical results were obtained with the 3 other anti-PfSUB1 mAbs (data not shown). Expression of MSP1, detected with mAb X509, was observed much earlier in schizont development, from the 2-3 nuclei stage. All images show the merged signals.

(E) Differential subcellular localisation of PfSUB1 and the microneme marker PfAMA1. Whilst both a rabbit anti-PfAMA1 antibody and mAb NIMP.M7 produce a punctate pattern typical of subcellular organelles, the signals do not superimpose, indicating that the proteins have different subcellular locations.

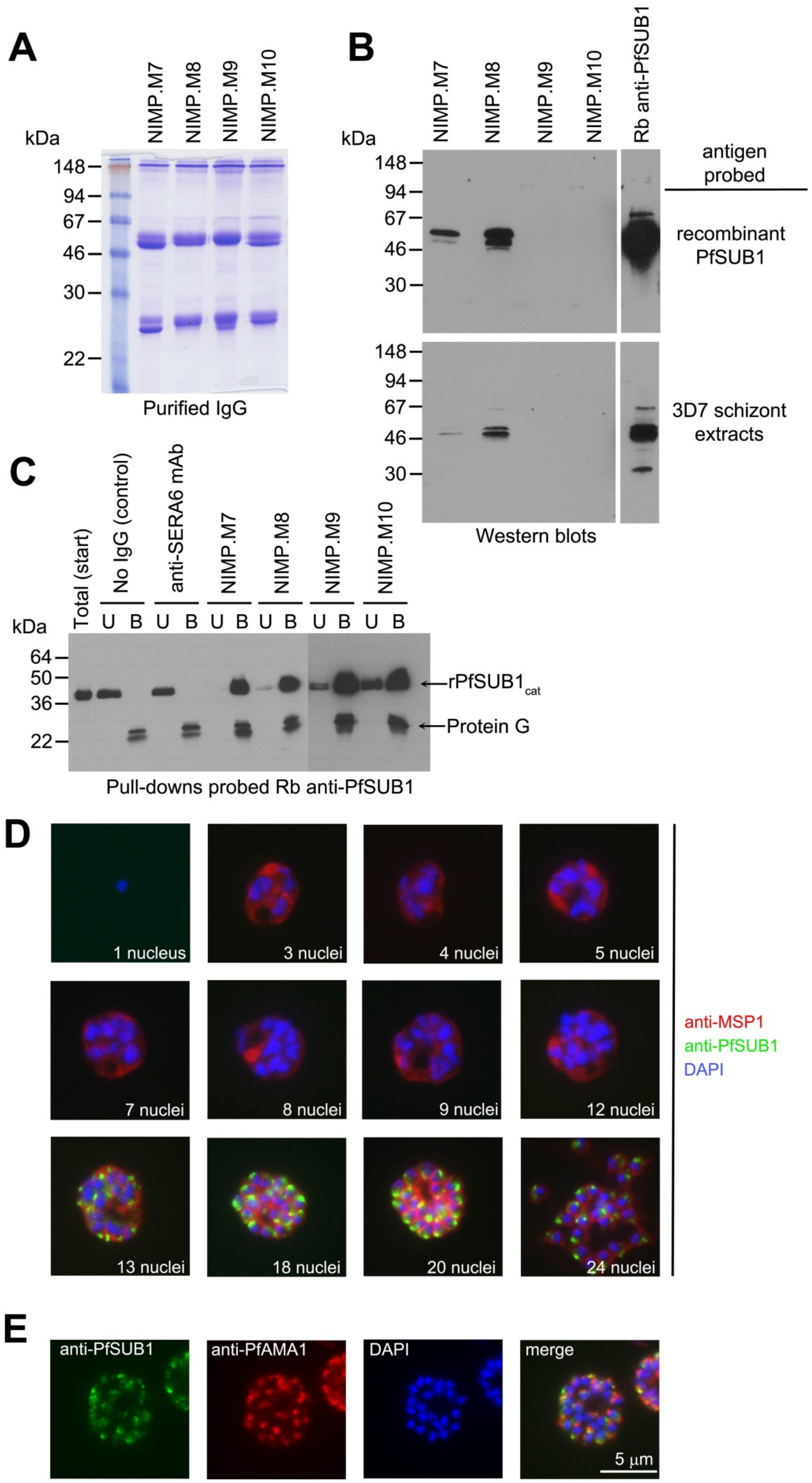
A
NIMP.M7
NIMP.M8
NIMP.M9
NIMP.M10
kDa
148
94
67
46
30
22
Purified IgG
B
NIMP.M7
NIMP.M8
NIMP.M9
NIMP.M10
Rb anti-PfSUB1
kDa
148
94
67
46
30
antigen probed
recombinant PfSUB1
3D7 schizont extracts
Western blots
C
Total (start)
No IgG (control)
anti-SERA6 mAb
NIMP.M7
NIMP.M8
NIMP.M9
NIMP.M10
U B U B U B U B U B U B
kDa
64
50
36
22
rPfSUB1cat
Protein G
Pull-downs probed Rb anti-PfSUB1
D
1 nucleus
3 nuclei
4 nuclei
5 nuclei
7 nuclei
8 nuclei
9 nuclei
12 nuclei
13 nuclei
18 nuclei
20 nuclei
24 nuclei
anti-MSP1
anti-PfSUB1
DAPI
E
anti-PfSUB1
anti-PfAMA1
DAPI
merge
5 μm

Figure S3. Discharge of PfAMA1 onto the surface of merozoites trapped in E64-stalled schizonts is associated with loss of PfSUB1 but retention of the host erythrocyte membrane

(A) IFA of wt 3D7 parasites allowed to develop beyond the point of egress in the presence of E64 only (50 μM). Schizonts displaying a merozoite-surface localisation of PfAMA1 (arrowed in the anti-PfAMA1 images) as a result of its discharge from micronemes, exhibit a very weak PfSUB1 signal compared to slightly less mature schizonts in which both the PfAMA1 and PfSUB1 signals remain punctate.

(B) IFA of E64-treated schizonts, double-stained with a mouse anti-PfAMA1 as well as a rabbit anti-human RBC antiserum. The presence of a residual host cell membrane surrounding parasites in which relocalisation of PfAMA1 to the merozoite surface has taken place (arrowed in the anti-PfAMA1 images), is evident. No loss of integrity of the RBC membrane was evident in any of a total of 35 such schizonts visually examined in which the RBC membrane was clearly delineated (i.e. not overlapping with or obscured by adjacent cells).

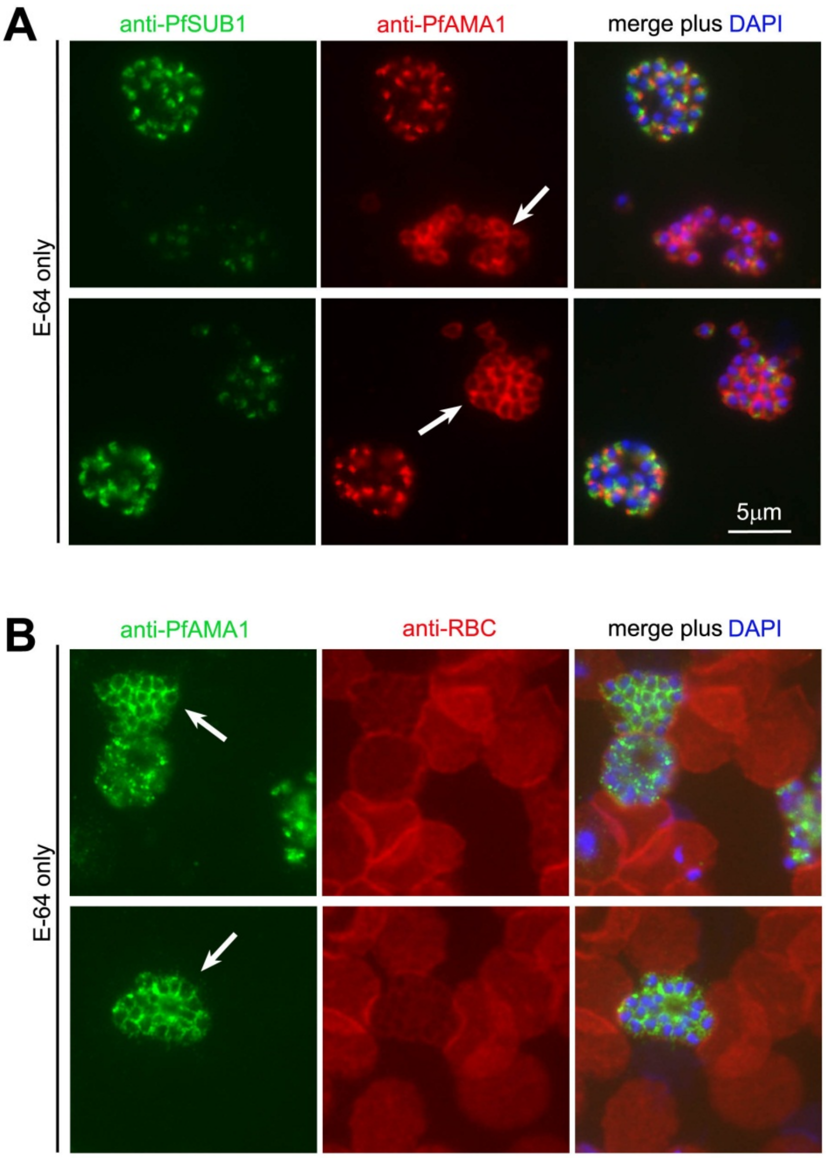
A
anti-PfSUB1
anti-PfAMA1
merge plus DAPI
E-64 only
5μm
B
anti-PfAMA1
anti-RBC
merge plus DAPI
E-64 only

Figure S4. Zaprinast-induced egress releases fully-processed SERA5 and is not blocked by C1 or C2 in the gatekeeper PfPKG$_{T618Q}$ parasite clone

(A) Western blot analysis of supernatant from zaprinast-treated schizonts (75 μM zaprinast, 20 min treatment) or a hypotonic extract of mature schizonts, prepared by freeze-thaw into a hypotonic buffer as described previously (Silmon de Monerri et al., 2011). Whereas the schizont hypotonic lysate contains predominantly full-length SERA5, as expected, only the fully processed P50 form of SERA5 is detectable in zaprinast-induced culture supernatants, showing that zaprinast-induced egress is associated with normal levels of PfSUB1 activity.

(B) Western blot detection of the P50 processed fragment of SERA5 in supernatants harvested from purified wt 3D7 schizonts or PfPKG$_{T618Q}$ schizonts following treatment as shown. Supernatants were harvested either at once or after 30 min incubation in the presence of the indicated additives. Zaprinast, C1 and C2 were used at concentrations of 75 μm, 2.5 μm and 1.5 μm respectively.

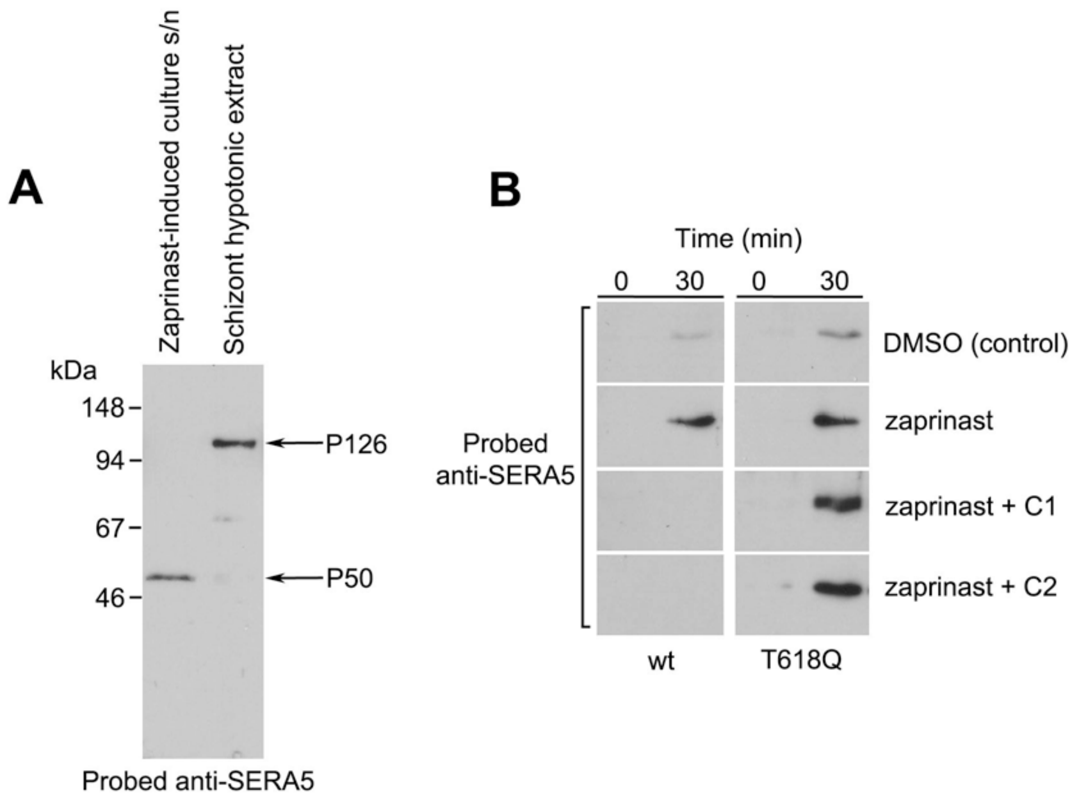

Figure S5. Zaprinast induces rapid discharge of PfAMA1 and PfSUB1

IFA analysis of mature schizonts treated for just 10 min with 75 μM zaprinast in the presence of E64 (50 μM) to prevent egress.

(A) Low magnification image to show the extent of the effects of zaprinast treatment. The surface translocation of PfAMA1 and decrease in the PfSUB1 signal (presumably due to its dispersal into the PV or loss due to PVM and RBC membrane poration) is easily evident. Examples of the unusual peripheral movement of micronemes and exonemes in immature schizonts are arrowed. Merged images include the DAPI staining to indicate parasite nuclei.

(B) Higher magnification image. An example of the unusual peripheral movement of micronemes and exonemes in an immature schizont is arrowed. Merged images include the DAPI staining to indicate parasite nuclei.

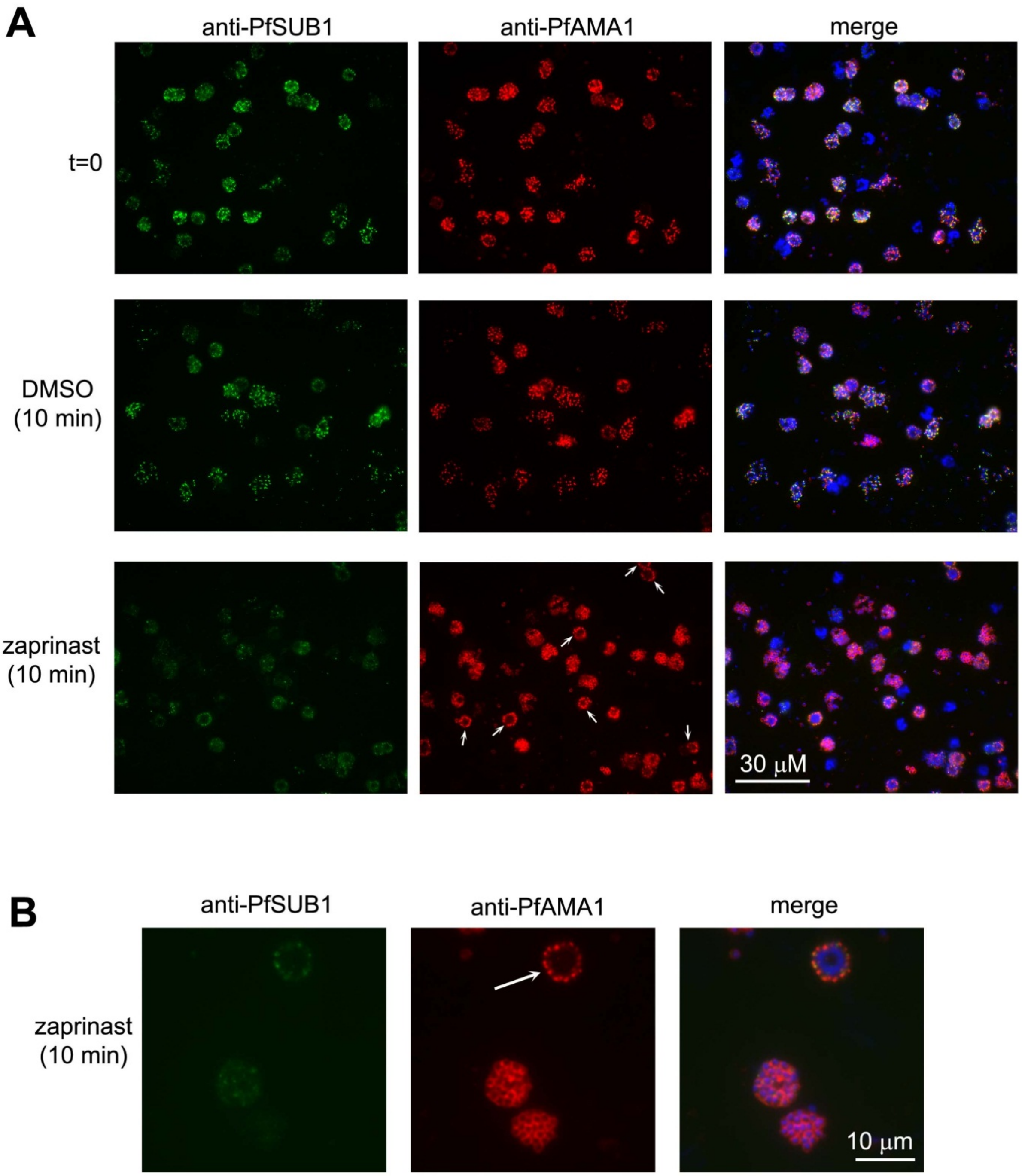
A
anti-PfSUB1
anti-PfAMA1
merge
t=0
DMSO
(10 min)
zaprinast
(10 min)
30 μM
B
anti-PfSUB1
anti-PfAMA1
merge
zaprinast
(10 min)
10 μm

Figure S6. Zaprinast preferentially induces egress of segmented schizonts

Percoll-enriched mature schizonts (~25% segmented; inset shows the distinction between a segmented and less mature schizont as observed by Giemsa stain) were supplemented with fresh RBC, divided into three flasks and incubated with DMSO only (0.5% v/v; control) or zaprinast (75 μM) for 50 min, then examined by microscopic analysis of Giemsa-stained thin films. Data are shown as mean values of triplicate microscopic counts (at least 500 RBC per count). Zaprinast primarily induced egress (loss) of the segmented schizonts (asterisked). Also, invasion in the control culture resulted in the appearance of ~5% new ring stage parasites over the culture period, whereas relatively few new rings appeared in the presence of zaprinast (also asterisked).

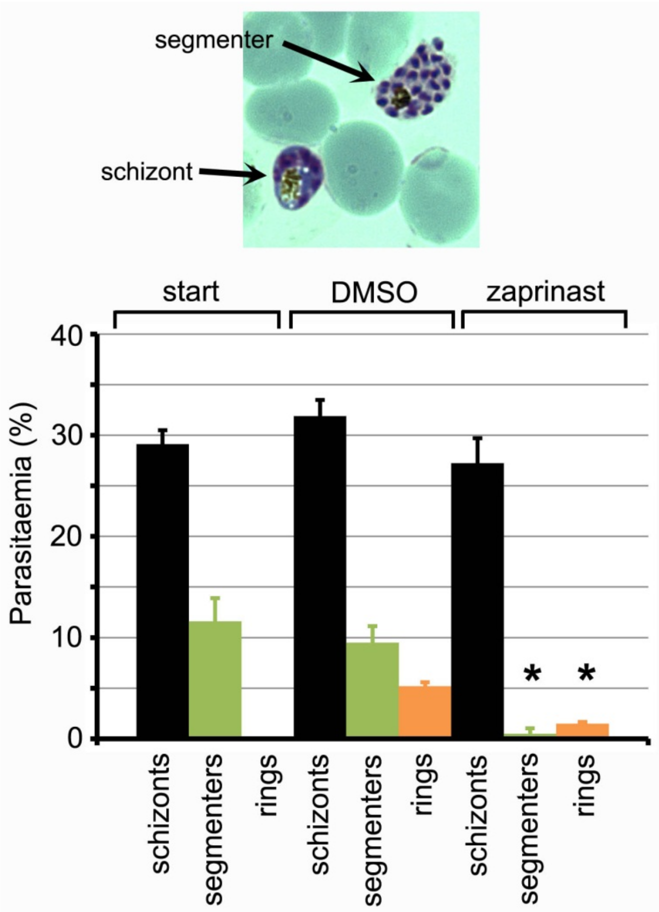

<u>Supplemental Movie legends</u>

Movie S1 Rapid egress of *P. falciparum* merozoites following release of a C1-mediated egress block

Schizonts allowed to mature for 7 h in the presence of the PfPKG inhibitor C1 were washed, resuspended in fresh warm medium without C1 and immediately observed by time-lapse video microscopy. Virtually complete egress is observed within 30 min.

Movie S2 Egress of *P. falciparum* merozoites following release of a C1-mediated egress block is prevented by the cysteine protease inhibitor E64

Schizonts allowed to mature for 7 h in the presence of the PfPKG inhibitor C1 were washed, resuspended in fresh warm medium without C1 but containing E64 (50 μM) and immediately observed by time-lapse video microscopy. In contrast to the cultures lacking E43 (Movie S1) no egress takes place over 30 min.

Movie S3 Egress in untreated mature schizonts

Cultures of purified schizonts (~25% segmented) were observed by time-lapse video microscopy for 30 min in the absence of zaprinast. Low levels of egress are evident.

Movie S4 Zaprinast induces egress

Cultures of purified schizonts (~25% segmented) identical to those in Movie S3 were observed by time-lapse video microscopy for 30 min in the presence of zaprinast (100 μM). Substantially increased levels of egress are evident compared to Movie S3.

Supplemental references

Blackman, M.J., Fujioka, H., Stafford, W.H., Sajid, M., Clough, B., Fleck, S.L., Aikawa, M., Grainger, M., and Hackett, F. (1998). A subtilisin-like protein in secretory organelles of Plasmodium falciparum merozoites. J Biol Chem *273*, 23398-23409.

Jean, L., Hackett, F., Martin, S.R., and Blackman, M.J. (2003). Functional characterization of the propeptide of Plasmodium falciparum subtilisin-like protease-1. J Biol Chem *278*, 28572-28579.

Silmon de Monerri, N.C., Flynn, H.R., Campos, M.G., Hackett, F., Koussis, K., Withers-Martinez, C., Skehel, J.M., and Blackman, M.J. (2011). Global identification of multiple substrates for Plasmodium falciparum SUB1, an essential malarial processing protease. Infect Immun *79*, 1086-1097.

Withers-Martinez, C., Saldanha, J.W., Ely, B., Hackett, F., O'Connor, T., and Blackman, M.J. (2002). Expression of recombinant Plasmodium falciparum subtilisin-like protease-1 in insect cells. Characterization, comparison with the parasite protease, and homology modeling. J Biol Chem *277*, 29698-29709.

Withers-Martinez, C., Suarez, C., Fulle, S., Kher, S., Penzo, M., Ebejer, J.P., Koussis, K., Hackett, F., Jirgensons, A., Finn, P.*, et al.* (2012). Plasmodium subtilisin-like protease 1 (SUB1): Insights into the active-site structure, specificity and function of a pan-malaria drug target. Int J Parasitol.

Yeoh, S., O'Donnell, R.A., Koussis, K., Dluzewski, A.R., Ansell, K.H., Osborne, S.A., Hackett, F., Withers-Martinez, C., Mitchell, G.H., Bannister, L.H.*, et al.* (2007). Subcellular discharge of a serine protease mediates release of invasive malaria parasites from host erythrocytes. Cell *131*, 1072-1083.
